# Priority-Aware Multi-Objective Task Scheduling in Fog Computing Using Simulated Annealing

**DOI:** 10.3390/s25185744

**Published:** 2025-09-15

**Authors:** S. Sudheer Mangalampalli, Pillareddy Vamsheedhar Reddy, Ganesh Reddy Karri, Gayathri Tippani, Harini Kota

**Affiliations:** 1 Manipal Institute of Technology Bengaluru, Manipal Academy of Higher Education, Manipal 560064, India; ms.sudheer@manipal.edu (S.S.M.); gayathri2.mitblr2024@learner.manipal.edu (G.T.); harini1.mitblr2024@learner.manipal.edu (H.K.); 2 Department of CSE (AIML), Keshav Memorial Engineering College, Hyderabad 500088, India; 3 School of Computer Science and Engineering, VIT-AP University, Amaravati 522241, India; ganesh.reddy@vitap.ac.in

**Keywords:** fog computing, task scheduling, simulated annealing (SA), multi-objective optimization

## Abstract

The number of IoT devices has been increasing at a rapid rate, and the advent of information-intensive Internet of Multimedia Things (IoMT) applications has placed serious challenges on computing infrastructure, especially for latency, energy efficiency, and responsiveness to tasks. The legacy cloud-centric approach cannot meet such requirements because it suffers from local latency and central resource allocation. To overcome such limitations, fog computing proposes a decentralized model by reducing latency and bringing computation closer to data sources. However, effective scheduling of tasks within heterogeneous and resource-limited fog environments is still an NP-hard problem, especially in multi-criteria optimization and priority-sensitive situations. This research work proposes a new simulated annealing (SA)-based task scheduling framework to perform multi-objective optimization for fog computing environments. The proposed model minimizes makespan, energy consumption, and execution cost, and integrates a priority-aware penalty function to provide high responsiveness to high-priority tasks. The SA algorithm searches the scheduling solution space by accepting potentially sub-optimal configurations during the initial iterations and further improving towards optimality as the temperature decreases. Experimental analyses on benchmark datasets obtained from Google Cloud Job Workloads demonstrate that the proposed approach outperforms ACO, PSO, I-FASC and M2MPA approaches in terms of makespan, energy consumption, execution cost, and reliability at all task volume scales. These results confirm the proposed SA-based scheduler as a scalable and effective solution for smart task scheduling within fog-enabled IoT infrastructures.

## 1. Introduction

The increasing adoption of Internet of Things (IoT) technologies has led to a revolutionary change in many areas, transforming the way devices communicate, create and process information [1]. As IoT applications expand into everyday life from smart homes and wearable technology to industrial automation and healthcare systems, the demand for smart, scalable and efficient computing infrastructure is increasing [2]. These interacting devices, ranging from sensors in industrial equipment to real-time health monitoring wearables, constantly generate huge amounts of heterogeneous data that require high bandwidth, low latency and real-time processing [3]. Of all the subsystems in the Internet of Multimedia Things (IoMT), the IoT world is the most unusual because it requires high-intensity computing, fast data transmission and enormous storage, in most cases, real-time video, audio and image data. The volume and intensity of multimedia data generated from IoMT-based applications are often used to test the responsiveness and capacity of traditional cloud-based systems and create latency and bandwidth issues that can impair free operation and user experience [4].

To overcome these shortcomings, cloud computing has long been used as a centralized model for providing scalable, virtualized resources over the Internet. It facilitates resource sharing, storage management, and on-demand computing [5] services required to handle large-scale data streams from the IoT. However, the centralized nature of cloud computing presents inherent disadvantages when faced with latency-demanding, location-based, and real-time applications [6]. For example, network-edge-generated data must reach centralized data centers for processing, which causes significant latency and reduces the quality of services [7]. Additionally, growing data security concerns, heavy network traffic, and wasteful bandwidth consumption further reduce the applicability of centralized cloud infrastructures to contemporary IoT needs. As IoT devices mature to enable next-generation applications with instant responsiveness and reliability, a new paradigm in architecture is needed—one that brings computational intelligence closer to the data source.

Fog computing acts as a vital paradigm to bridge the gap between cloud data centers and edge devices. By bringing cloud features to the network’s edge, fog computing makes it possible to handle computing, storage, and networking near the devices that create data [8]. This decentralized approach not only reduces data transmission latency but also relieves bandwidth pressure at the core network and increases the responsiveness of latency-intensive applications. In this context, fog nodes—typically lightweight servers, routers or gateways—act as intermediate processing units that allow for local decision-making and task execution [9]. This proximity to data sources enables fog computing to effectively support real-time applications such as augmented reality, industrial automation, autonomous driving and remote health diagnostics. In addition to latency minimization, fog computing allows for increased data security and privacy for transmitting sensitive data to centralized points, which is critical for applications that handle personal or critical infrastructure data [10,11].

However, the dynamic characteristics of a fog environment and the decentralization impose new challenges, particularly in task scheduling. Efficiently assigning and executing computational jobs across geographically dispersed and resource-constrained fog nodes is no small task. IoT tasks are typically represented as directed acyclic graphs (DAGs), where each node represents an individual computer task and an edge represents task dependency. In these representations, scheduling means mapping tasks onto appropriate fog nodes without violating their execution order and resource requirements. The key goals of task scheduling using fog are to minimize makespan (the sum of the time it takes for all tasks to complete reduce energy), optimize operational cost, and ensure high reliability and fault tolerance. These goals are typically interdependent and must be traded off to achieve maximum system performance.

**Figure 1:**
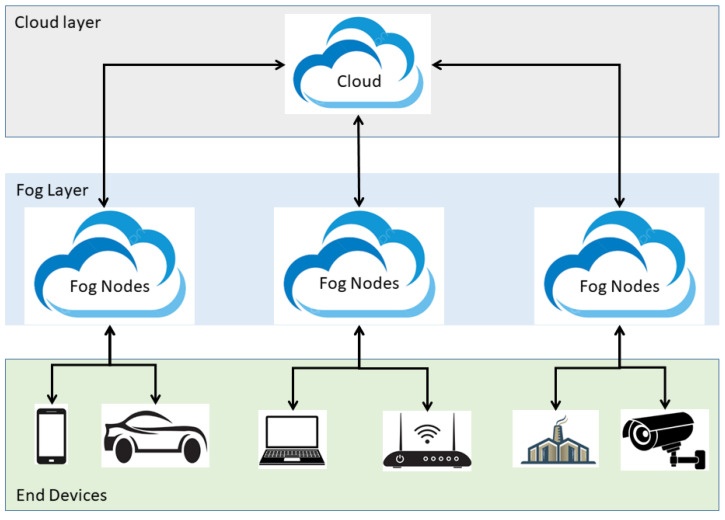
Fog computing architecture.

Figure 1 describes the fog computing architecture. The layers of fog computing architecture are:

- **IoT/End Devices Layer (Edge Layer):** Comprises sensors, actuators, smart devices, and mobile devices that generate raw data. Performs data collection and can do preliminary filtering or processing. Sends data upstream to fog nodes for further processing.
- **Fog Layer (Intermediate Layer):** Consists of fog nodes deployed near the network edge, such as gateways, routers, micro data centers, or edge servers. Responsible for local data processing, storage, and analytics, reducing latency and network congestion. Supports real-time decision making, task offloading, and context-aware services. Communicates with both the IoT layer and cloud layer.
- **Cloud Layer (Central Layer):** Centralized, large-scale data centers for long-term storage, heavy computation, and advanced analytics. Handles non-latency-sensitive tasks that require high processing power. Acts as a backup and coordination layer for fog nodes.

Despite the advantages of fog computing, efficient task scheduling remains a major challenge due to fluctuating workloads, heterogeneous resources, limited energy availability, and the need to handle task priorities in real time. Task scheduling is an NP-hard problem because of the increasing number of devices and dynamic operational environments. Traditional heuristic and metaheuristic methods often optimize only a single objective or fail to adapt under dynamic conditions; although they are computationally efficient, they often fail to adapt to real-time demands and balance multiple performance metrics, leading to poor resource utilization and degradation of QoS. To overcome this, we propose a simulated annealing (SA)-based multi-objective optimization framework for fog task scheduling. SA, a probabilistic technique inspired by metallurgical annealing, is effective in exploring large solution spaces and avoiding local optima. Our approach optimizes a composite objective function that includes makespan, energy consumption, cost, and reliability. A priority-based penalty mechanism is included to ensure that urgent tasks are not delayed by less critical ones, which improves system responsiveness. The algorithm starts with an initial task-node mapping and iteratively explores neighboring schedules. Each configuration is evaluated using a cost function, and new schedules are accepted based on temperature-controlled probabilities, balancing exploration, and convergence. Gradual cooling of the schedule allows for stable, nearly optimal solutions to be found.

### 1.1. Motivation and Key Challenges

#### 1.1.1. Motivation

The boom in IoT devices has led to big changes in computing approaches. It has brought challenges like handling huge data and meeting instant processing demands. Cloud systems have become key players because they offer the capacity to meet the fast response and dependability needs of time-sensitive IoT applications. Fog computing, on the other hand, brings computing closer to the edge. This approach lowers delays and reduces data use. However, distributing tasks this way creates issues. These include changes in resource availability, different node abilities, and unpredictable task loads. To tackle this, there is a strong need to create scheduling methods. Such methods should use the closeness of fog nodes to provide faster, more dependable, and energy-friendly task execution.

#### 1.1.2. Challenges

To schedule tasks in fog environments, planners need to tackle multiple challenges and aim to achieve goals like shorter makespan, lower energy use, cost reductions, and higher system reliability. Many existing methods fail because they depend on fixed techniques or aim at narrow goals. This issue gets worse when task priorities change or node conditions shift. Handling task scheduling in fog networks is NP-hard. Traditional algorithms do not perform in large systems. The need to assign task priorities complicates things further, as it requires prioritizing heavy tasks while ensuring fairness. Edge nodes with limited power face significant challenges in staying energy efficient. Keeping operational costs low is also necessary to sustain fog infrastructure in the long run. Handling fault tolerance and keeping nodes reliable makes scheduling harder. This situation creates the need to adopt systems capable of managing potential failures. Shifting computing closer to the network’s edge helps lower delays and reduce data usage. However, this distributed setup creates challenges with resource availability that changes, differences in how capable nodes are, and varying workloads. These challenges highlight the demand to develop better scheduling methods. These methods can take advantage of the nearby fog nodes to provide quick, dependable, and energy-saving task performance.

### 1.2. Key Contributions

- We proposed a priority-aware, multi-objective task scheduling framework for fog–cloud environments. This framework explicitly integrates urgent task responsiveness into the scheduling process, which is often overlooked in prior methods.
- We developed a Simulated Annealing (SA)-based scheduling algorithm that incorporates priority-aware penalties. Unlike GA- or RL-based schedulers, this approach balances cost and makespan while improving responsiveness for critical tasks.
- The Simulated Annealing algorithm is tested against the GoCJ dataset (task sizes between 15,000 and 900,000 units), ensuring the evaluation against realistic and varied workloads found in fog–cloud environments.
- The proposed SA method is systematically compared against well-known scheduling frameworks, including ACO, PSO, I-FASC, and M2MPA. This allows us to demonstrate how the proposed model addresses their limitations, particularly in handling urgent tasks.

The outline of the paper is condensed as follows: Section 2 reviews the related literature relevant to the proposed method. Section 3 describes the problem formulation of the proposed work. Section 4 illustrates the working procedure of Simulated Annealing for Task Scheduling. Section 5 reports the experimental results and provides a detailed discussion based on performance metrics, and finally, Section 6 concludes the paper, highlighting key findings and suggesting directions for future work.

## 2. Related Work

The authors of [12] examine how to schedule tasks in fog-based IoT apps to cut down on service delays and energy expenses while meeting task deadlines. The researchers introduce a new way to schedule tasks called Fuzzy Reinforcement Learning (FRL), which combines fuzzy control and reinforcement learning techniques. The rise in IoT devices has brought big changes to computing models, leading to huge amounts of data and the need for quick processing. Cloud systems play a key role by handling the fast-response and high-reliability demands of time-sensitive IoT applications. Fog computing provides a flexible solution by bringing computing power closer to the edge to reduce delay and lower data usage. They build a mixed-integer nonlinear programming (MINLP) model to minimize the energy consumed by fog resources and the time required to complete tasks. The approach also considers deadlines and resource limitations. To manage a large number of tasks in changing environments, they use a fuzzy-based reinforcement learning technique. This method starts by sorting tasks with fuzzy logic, focusing on things like deadlines, data sizes, and instruction counts. After that, they apply an on-policy reinforcement learning scheduling method called SARSA learning, which has a stronger influence on long-term rewards compared to Q-learning. The results reveal that their FRL method performs better than other algorithms, leading to a 23% improvement in service latency and an 18% increase in energy costs. This method handles service time and energy use, ensuring deadlines are met to maximize resource use in fog computing setups.

The authors of [13] suggest an effective load-balancing system specifically tailored for resource scheduling in cloud-based communication systems, focusing mainly on healthcare applications. The authors tackle the increasing need for real-time data processing in healthcare systems, where the most important phase is the allocation of resources on time. In order to overcome inefficiencies like unbalanced load assignment, absence of fault tolerance, and higher latency in current methods, the current framework employs RL and genetic algorithm to dynamically assign the resources. Agent-based models are used by the system for assigning resources and scheduling tasks with the goal of minimizing make-span, throughput, and latency. According to experimental tests, the current framework has been shown to outperform other approaches.

The authors of [14] address the intricate issue of scheduling Bag-of-Tasks (BoT) applications in fog-based Internet of Things (IoT) networks by developing a multi-objective optimization strategy. The core objective is to achieve an optimal balance among three inherently conflicting performance metrics: minimizing execution time (makespan), reducing operational costs, and enhancing system reliability. The makespan criterion focuses on ensuring timely task completion, while cost optimization considers the monetary expenses associated with CPU, memory, and bandwidth usage. Reliability is quantified through a novel metric, the Scheduling Failure Factor (SFF), which reflects the historical failure tendencies of fog nodes based on Mean-Time-To-Failure (MTTF) data.

The authors of [15] proposed a hybrid approach, combining an FIS with a GA to address multi-objective optimization and condition satisfaction problems. The proposed model integrates sequencing, scheduling algorithms, and partitioning to optimize the trade-off between the users and resource providers, such as cost, resource utilization, and makespan. The FIS performs scheduling based on constraints like budget and deadline, while the GA optimizes task allocation by resource usage and reducing cost and makespan. The experiments are implemented using workflows, and the results are outperformed in terms of makespan by reducing to 48% compared with EM-MOO and 41% compared with MAS-GA. Additionally, the process ensures better compliance with user-defined deadlines and budget constraints.

The author [16] introduces a novel approach for cyber-physical-social systems (CPSSs), utilizing a modified version of the MPA, referred to as M2MPA. The main task is to minimize the energy consumption and makespan during the processing of IoT tasks offloaded to fog nodes. The authors argue that the existing metaheuristic-based schedulers and traditional techniques are insufficient for solving this NP-hard problem with high precision in an appropriate time. To address this, M2MPA incorporates two key improvements over the classical MPA: changing the fish aggregating devices (FAD) mechanism with a polynomial crossover operator to enhance examination and using an adaptive CF parameter to improve exploitation.

The authors of [17] proposed an improved task scheduling method called I-FASC (Improved Firework Algorithm-based Scheduling and Clustering) for fog computing environments. The authors address the challenges of minimizing the processing time and check balanced load distribution across fog devices, which are critical due to the weak processing and limited resource capabilities of fog nodes compared to cloud servers. To achieve this, they introduce an enhanced version of the Firework Algorithm (I-FA), incorporating an explosion radius detection mechanism to prevent optimal solutions from being overlooked. The I-FASC method includes task clustering based on likely storage space, completion time, and bandwidth requirements, enabling targeted resource scheduling. Resources in the fog layer are categorized into three types: storage, computing, and bandwidth, and allocated according to their respective capacities. The fitness function measures both time and load values, with weights tuned to focus on either execution time or load balance according to the optimization target.

The author [18] proposes FLight, for federated learning (FL) on the edge- and fog-computing platforms. The authors generalize the FogBus2 framework to develop FLight, incorporating new machine learning (ML) models and accommodating mechanisms for access control, worker selection, and storage implementation. The framework is intended to be conveniently extensible, with the capability to add different ML models as long as import/export and merging of model weights are specified. FLight also accommodates asynchronous FL and provides flexibility in storing model weights on different media, which eases deployment across various computing resources. The paper mentions two worker selection algorithms presented in FLight to enhance training time effectively. These algorithms emphasize quicker workers while increasingly the utilization of slower ones in subsequent rounds of training. This approach balances accuracy improvement with training time efficiency, avoiding the pitfalls of excluding slower workers entirely or over-relying on them. The authors also discuss the trade-offs between energy consumption, model accuracy, and time efficiency, noting that previous research often focused on accuracy-energy trade-offs without fully considering time efficiency.

The authors [19] introduce a novel EEIoMT. The aim of EEIoMT is to reduce latency, reduce energy consumption, and enhance the QoS for IoMT applications. It is specially suited for monitoring the patient data to raise the quality of life by reducing medical expenses. The framework categorizes tasks into three categories (normal, moderate, and critical) based on the requirement for efficient scheduling by reducing energy consumption. The proposed framework utilizes the ifogsim2 simulator, and results demonstrated that the EEIoMT significantly reduces energy consumption, network utilization, and response time to existing methods. However, authors did not consider important parameters like cost and reliability for efficient scheduling.

The authors of [20] address the challenge of task scheduling in fog computing environments, proposing an optimized solution using an ant colony algorithm to achieve multi-objective goals such as minimizing makespan, improving load balancing, and reducing energy consumption. The authors are specifically interested in Directed Acyclic Graph (DAG) problems, which are prevalent in most applications involving effective resource allocation and processing. The method proposed starts with the initialization of pheromone levels on all possible routes, with ants deployed randomly on these routes to search for potential solutions. Each ant computes transition probabilities from heuristic information and pheromone trails, updating the levels of the pheromones after every iteration in order to steer subsequent searches towards better solutions. This is how ants solve the shortest path to food using positive feedback processes and converge towards near-optimal solutions for the NP-hard DAG task scheduling problem.

The authors of [21] introduced an EDLB framework, which combines MPSO and CNN for the limitations of existing load balancing methods. The framework is composed of three modules: Resource Monitor(FRM), CNN-Base classifier (CBC) and Optimized Dynamic Scheduler (ODS) to achieve real-time load balancing. Frm is used to monitor the resources and store the data, CBC calculates the fitness function of the servers and ODS schedules tasks dynamically by optimizing resource utilization and latency. EDLB evaluation results are better than the GA, BLA, WRR, and EPSO in terms of cost, makespan, resource utilization and load balancing. However, the authors did not consider impact parameters like reliability and energy consumption to improve the efficiency of scheduling.

The primary aim of [22] is to enhance real-time data processing and reduce latency for critical healthcare tasks by leveraging fog computing’s proximity to end-users. HealthFog uses fog nodes to process and analyze cardiac patient data locally, minimizing reliance on cloud resources and improving overall efficiency. The proposed framework consists of three main components: workload management, resource arbitration, and deep learning modules. The workload manager monitors task queues and job requests, while the arbitration component assigns fog or cloud resources for optimal load balancing. The deep learning module utilizes patient datasets for training, testing, and validation in a 7:2:1 ratio, enabling automated analysis of cardiac data. By deploying HealthFog within the FogBus framework, the system achieves seamless integration with IoT-edge-cloud environments, ensuring real-time data processing. The authors identify a number of the benefits of fog computing compared to conventional cloud-based systems, including lower data movement, removal of bottlenecks due to centralized systems, reduced data latency, location awareness, and enhanced security for encrypted data. These capabilities render fog computing well-suited to time-sensitive healthcare applications, like continuous patient monitoring.

The challenge of providing accurate execution of tasks in fog computing systems is addressed by the authors of [23] through the presentation of a learning-based primary-backup approach named ReLIEF (Reliable Learning based In Fog Execution). The authors concentrate on enhancing the reliability of running tasks within fog nodes with deadlines and reducing delays. The suggested strategy comes into play especially for applications that demand low-latency processing near the data source. ReLIEF provides a framework for choosing both primary and secondary fog nodes for task processing. Contrary to conventional methods where a task is allocated to one single fog node without redundancy, this method provides fault tolerance by allocating a standby node to process tasks in case the primary one fails or encounters delays. The selection process uses machine learning algorithms to estimate the most appropriate primary and backup nodes according to capabilities such as computational power, network status, and past performance data. The paper compares ReLIEF with three cutting-edge approaches: DDTP (Dynamic Deadline-aware Task Partitioning), MOO (Multi Objective Optimization), and DPTO (Deadline and Priority aware Task Offloading). Experimental results show that ReLIEF performs better than these algorithms on reliability, with a greater percentage of tasks completing their deadlines even in the face of fluctuating workload levels and resource limitations. In particular, the strategy performs well in high uncertainty or unforeseen environmental conditions, including sudden hikes in network latency or short-term unavailable fog nodes.

From Table 1, existing research on task scheduling, ACO and PSO algorithms are metaheuristic approaches used for global search and task allocation. However, they often suffer from premature convergence and lack of ability to adapt dynamic fog–cloud workloads, which reduces their applicability in latency-sensitive environments. Frameworks like I-FASC and M2MPA are important for fog scheduling, but their focus is mainly on cost efficiency and throughput, and they do not address the priority handling or urgent task responsiveness, both of which are critical in fog computing. Recent research incorporating reinforcement learning and deep learning is adaptive, but suffers from high computational overhead. Hybrid fog–cloud models utilize fog for latency-critical tasks and cloud for compute-intensive tasks, but it is difficult to balance workload against reliability. To solve these issues, our Simulated Annealing (SA) model incorporates a priority-aware penalty mechanism that balances cost and makespan while also ensuring responsiveness to urgent tasks. Therefore, while ACO, PSO, I-FASC, and M2MPA are valuable baselines, their limitations in addressing urgency and priority-awareness highlight the necessity of our SA-based solution.

## 3. Problem Formulation

**Figure 2:**
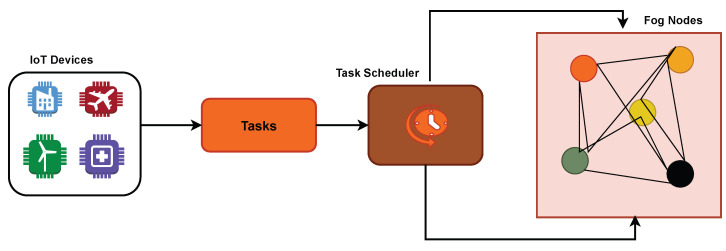
Proposed architecture.

Figure 2 shows the proposed architecture of the priority-aware multi-objective task scheduling framework in fog computing. In the architecture, there are three major components: **Input Layer:** Tasks generated from IoT/IoMT devices are collected, each having parameters such as execution time, reliability requirement, energy demand, cost factor, and priority level. These inputs form the basis for scheduling decisions.

**Scheduling Core:** At the heart of the framework lies the Simulated Annealing (SA)-based scheduler. This module will explore the task-to-node mapping space using a balancing exploration, probabilistic search strategy, and exploitation. The optimization works through a combined cost function. It ties together four main goals: makespan, energy use, execution cost, and reliability. A priority-focused penalty gets used to make sure important tasks go to nodes that are less crowded and more dependable. Each part of the architecture matches up with specific math formulas (Equations (1)–(7)), which are laid out in the problem description. This connects the theoretical aspects with how the system functions.

**Execution & Feedback Layer:** After deciding on schedules, the system runs tasks across fog nodes. It tracks metrics such as how long tasks take, how work is shared, and how resources are used. These results go back to the scheduler, allowing the task allocation process to improve through repeated adjustments.

Within the context of fog computing systems where many tasks are being scheduled across a distributed collection of edge and fog nodes, it is necessary to integrate multi-objective optimization techniques that account for task-specific properties like execution time, energy usage, cost, node reliability, and most importantly, task priority. Task priority is instrumental in making the system responsive and efficient, especially for time-critical or mission-critical applications. Each task $T_{i}$ is assigned a priority level $p_{i}\in \left\{1,2,\ldots ,10\right\}$, such that higher values of pi represent higher urgency. The objective of the scheduler is to allocate the high-priority jobs to less-loaded and more reliable nodes to reduce execution delays and minimize resource contention. The Simulated Annealing (SA) algorithm is used as the fundamental optimization paradigm, able to navigate intricate scheduling spaces and escape local minima. For the purpose of directing the optimization, a composite cost function is specified, with four primary performance measures: energy usage, execution cost, makespan, and a priority-aware penalty on tasks. The makespan *M* is the maximum completion time among all fog nodes and reflects the overall system latency. It is formally defined as:

(1) $$M=\max_{j\in \{1,\ldots ,N\}}\left(\sum_{i=1}^{T}d_{i}\cdot I\left(n_{i}=j\right)\right)$$

where $d_{i}$ is the execution duration of task $T_{i}$, $n_{i}$ is the index of the node assigned to $T_{i}$, *N* is the total number of fog nodes, and I(·) is the indicator function, which equals 1 if the condition inside holds true and 0 otherwise. This approach makes sure tasks get spread out so all nodes have a similar workload, cutting down the time needed to finish the whole schedule.

Total energy consumed, *E*, is determined by summing up the amount of energy each activity consumes while running. Assuming each fog node consumes power at a constant rate P (watts), the total energy consumption for all activities resembles this:

(2) $$E=\sum_{i=1}^{T}d_{i}\cdot P$$

This measure, expressed in joules or kilojoules, has an important role in situations where energy resources are scarce such as in green computing scenarios or devices powered by batteries. The running cost *C*, is calculated by taking the time it takes to complete a task and multiplying it by a unit cost rate, ρ. The unit cost rate represents the monetary cost per unit of computation time:

(3) $$C=\sum_{i=1}^{T}d_{i}\cdot \rho$$

This concept lets users schedule while considering economic factors. This becomes important when fog resources charge based on the amount of computation used.

The system’s reliability *R* is defined as the likelihood that all planned tasks finish without any issues. If we assume each node has a reliability factor r∈[0,1], and if we consider the nodes to be independent, the reliability for carrying out all *T* tasks becomes:

(4) $$R=\prod_{i=1}^{T}r=r^{T}$$

This product assumes all nodes are reliable to keep things simple. However, it can be extended to work with different environments where each node has its own reliability $r_{i}$.

To integrate task priority explicitly into the optimization model, a priority penalty function $P_{p}$ is introduced. This penalty discourages assigning high-priority tasks to heavily loaded nodes, which would result in increased waiting time and QoS degradation. The priority penalty is defined as:

(5) $$P_{p}=\sum_{i=1}^{T}p_{i}\cdot W_{n_{i}}$$

where $W_{n_{i}}$ is the total workload on the node $n_{i}$ to which task $T_{i}$ is assigned. The workload $W_{j}$ on node *j* is computed as:

(6) $$W_{j}=\sum_{k=1}^{T}d_{k}\cdot I\left(n_{k}=j\right)$$

where Equation (6) computes the workload of node j by adding up the demands ($d_{k}$) of all tasks that are mapped to it. The indicator function $I(n_{k}=j)$ guarantees that only tasks mapped onto the node are added.

The introduction of energy, cost, reliability, and the priority aware penalty into the optimization model is driven by realistic constraints of fog computing. Equation (2) accounts for limited power budgets of fog nodes; the cost of execution Equation (3) captures the economic viability of using resources such as reliability; Equation (4) provides assurance against failures in heterogeneous nodes; and the priority penalty Equation (5) ensures responsiveness towards time-critical tasks. Together, these metrics meet the core QoS objectives of fog-enabled IoT systems and thus make the optimization framework theoretically sound and practically feasible.

This penalty ensures that tasks with higher $p_{i}$ values (more urgent tasks) significantly increase the cost when scheduled on overloaded nodes, encouraging the scheduler to favor lightweight and fast nodes for important tasks.

Bringing together all the above components, the total cost function used for Simulated Annealing becomes:

(7) $$\mathrm{Total}\;\mathrm{Cos}t=\alpha M+\beta E+\gamma C+\delta P_{p}$$

Here, $\alpha ,\;\beta ,\;\gamma ,\;\delta \in R^{+}$ are tunable weights that determine the importance of each metric in the optimization process. These weights allow the scheduler to prioritize different objectives depending on the application context, for instance, assigning greater weight to δ in scenarios where task urgency dominates.

While optimizing, the Simulated Annealing method looks at the solution space by creating fresh schedule options by reassigning tasks to different nodes. If the new schedule $S^{\prime }$ ends up with a total cost smaller than the current schedule *S*, it gets accepted. If not, it might still be accepted based on a probability determined by the Boltzmann distribution.

(8) $$P_{\mathrm{accept}}=\left\{\begin{array}{cc} 1 & \mathrm{if}\;\Delta \mathrm{Cost}\leq 0\\ \exp\left(-\frac{\Delta \mathrm{Cost}}{T}\right) & \mathrm{if}\;\Delta \mathrm{Cost}>0 \end{array}\right.$$

where $\Delta \mathrm{Cost}=\mathrm{Cost}\left(S^{\prime }\right)-\mathrm{Cos}t\left(S\right)$, and *T* represents the current temperature in the annealing schedule, which falls over time. Algorithm 1 can optimize and converge toward an optimal or nearly optimal schedule when the temperature drops because there is less chance of accepting subpar solutions. By adding task priority to the multi-criteria cost model. This model ensures that the scheduler can adapt dynamically to crucial application demands without sacrificing system wide efficiency, in addition to makespan, energy, cost, and reliability. The result is a robust scheduling framework suitable for the latency-sensitive and heterogeneous characteristics of modern fog computing systems.
**Algorithm 1** Priority-Aware Task Scheduling in Fog Computing**Require:**       $T=\{T_{1},T_{2},\ldots ,T_{n}\}$: Set of tasks       $P=\{p_{1},p_{2},\ldots ,p_{n}\}$: Priority of each task (1 to 10; higher = higher priority)       $D=\{d_{1},d_{2},\ldots ,d_{n}\}$: Execution time for each task       $N=\{N_{1},N_{2},\ldots ,N_{k}\}$: Set of fog nodes**Ensure:**       schedule[$T_{1}\ldots T_{n}$]: Final task-to-node assignments       workload[$N_{1}\ldots N_{k}$]: Workload per node after assignment      1:Initialize workload[$N_{1}\ldots N_{k}$] ←0      2:Initialize schedule[$T_{1}\ldots T_{n}$] ←−1      3:**for** each task $T_{i}$ in *T* **do**      4:      best_node← None      5:      min_penalty←∞      6:      **for** each node $N_{j}$ in *N* **do**      7:            projected←workload[$N_{j}$] +D[i]      8:            $penalty\leftarrow p_{i}\times projected$      9:            **if** penalty<min_penalty **then**    10:                  min_penalty←penalty    11:                  $best\text{\_}node\leftarrow N_{j}$    12:            **end if**    13:      **end for**    14:      Assign $T_{i}$ to best_node    15:      workload[best_node] += D[i]    16:      schedule[$T_{i}$] ←best_node    17:**end for**

The time complexity of the priority-aware task scheduling algorithm in Fog Computing is O(n.k), where n denotes the number of tasks, and k denotes the number of fog nodes. In the first stage, workload and schedule arrays take the time of O(n + k) from the initialization part. The outer loop from lines 3–17 runs with n iterations. The inner loop that runs from lines 6–13 takes O(k) as it needs to find the best assignment of a task to be chosen from the available k nodes. The total time complexity is calculated as O(n + k) + O(n.k). The term n.k dominates for larger inputs. Therefore, the time complexity for the above approach is O(n.k).

## 4. Simulated Annealing Task Scheduling

**Figure 3:**
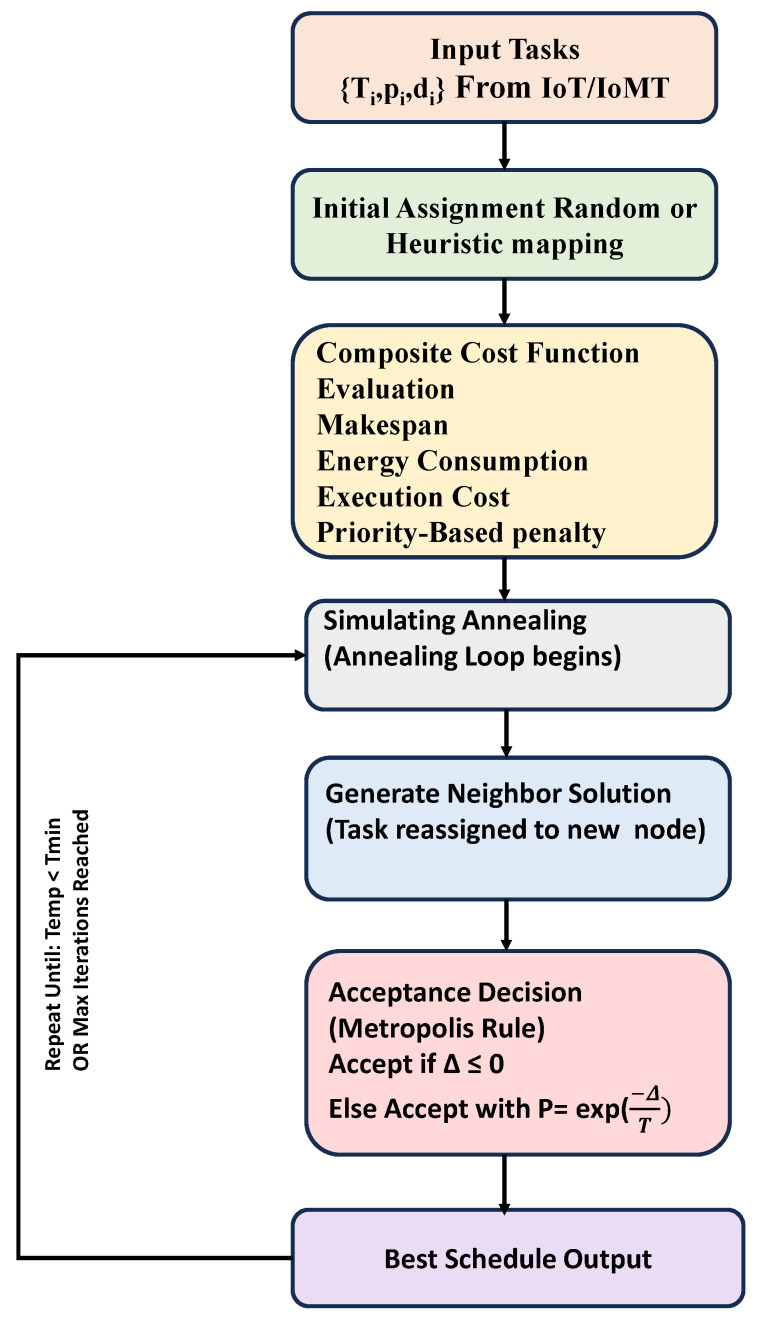
Proposed work flowchart.

The Simulated Annealing Figure 3 begins by making a random task plan for nodes. A cost function then evaluates this plan. It considers things like task completion time, energy usage, execution cost, and a penalty tied to priority. After that, the algorithm tweaks the plan with small changes called neighbors. Sometimes, it even chooses worse options based on probability linked to temperature. This helps it escape local optima and work toward a near-optimal setup that balances the main goals. The method continues to run a set number of times or stops when the temperature drops under a specific limit. At that moment, it returns the best schedule found.

Unlike GA-based schedulers [15] and RL-based methods [12,13], which primarily focus on cost and energy, our SA-based model incorporates priority-aware penalties to improve responsiveness for critical tasks. Previous models such as I-FASC [17] and EEIoMT [19] do not consider urgent task responsiveness, whereas our method integrates the urgency and priority-awareness within a multi-objective formulation, positioning it as an advancement over these existing approaches.

### 4.1. Initialization

To begin the Simulated Annealing (SA) process for task scheduling, we first formalize a schedule σ as a mapping

(9) $$\sigma^{\left(0\right)}:\left\{1,\ldots ,T\right\}\to \left\{1,\ldots ,N\right\}$$

where each task i∈{1,…,T} is assigned to a fog node $\sigma^{\left(0\right)}\left(i\right)\in \left\{1,\ldots ,N\right\}$. In its simplest form, this initial schedule is generated uniformly at random:

(10) $$\mathrm{Pr}\left(\sigma^{\left(0\right)}\left(i\right)=j\right)=\frac{1}{N},\;\mathrm{for}\;j=1,\ldots ,N$$

ensuring that every possible assignment has equal probability. This purely stochastic start helps the algorithm cover the entire solution space without bias, promoting broader exploration in early iterations.

However, uniform random seeding can be augmented by heuristic or hybrid strategies to improve convergence speed. For example, one might pre-assign high-priority tasks to the least-loaded nodes using a simple greedy rule, or incorporate domain knowledge by placing tasks with the longest durations on more powerful nodes. Such heuristic seeding yields an initial $\sigma^{\left(0\right)}$ closer to the optimum, reducing the number of uphill moves required during the annealing process.

Once $\sigma^{\left(0\right)}$ is fixed, we compute its initial cost via the multi-objective function:

(11) $$\mathrm{Cost}\left(\sigma^{\left(0\right)}\right)=\alpha M\left(\sigma^{\left(0\right)}\right)+\beta E\left(\sigma^{\left(0\right)}\right)+\gamma C\left(\sigma^{\left(0\right)}\right)+\delta \mathrm{Priority}\left(\sigma^{\left(0\right)}\right)$$

This value populates both the current cost and best-so-far cost:

(12) $${\mathrm{Cost}}_{\mathrm{current}}={\mathrm{Cost}}_{\mathrm{best}}=\mathrm{Cost}\left(\sigma^{\left(0\right)}\right)$$

Maintaining these two references allows SA to track progress and preserve the most promising schedule encountered.

A critical SA hyperparameter is the initial temperature $T_{0}$. If chosen too low, the algorithm behaves like a greedy hill-climber and easily becomes trapped in local minima; if too high, it wastes time exploring dominated regions. A common approach is to estimate $T_{0}$ based on the cost landscape of random samples. Specifically, by generating *S* random schedules ${\left\{\sigma^{\left(s\right)}\right\}}_{s=1}^{S}$, computing their pairwise cost differences $\Delta_{s}$, and taking the sample standard deviation $\sigma_{\Delta }$, one sets

(13) $$T_{0}=-\frac{\sigma_{\Delta }}{\ln(p_{0})}$$

where $p_{0}\in \left(0.5,0.9\right)$ is a target initial acceptance probability (e.g., $p_{0}=0.8$). This principled choice ensures that a high fraction of uphill moves are accepted at the start, facilitating wide-ranging exploration.

Finally, before entering the main annealing loop, practitioners often precompute cost components (e.g., node workloads, per-task energy) and initialize logging structures to record metrics such as cost, temperature, and acceptance rates. This rich initialization phase—combining random or heuristic seeding, informed temperature selection, and systematic bookkeeping—lays a robust foundation for the subsequent SA iterations, dramatically improving both the efficiency and the quality of the final task schedule.

### 4.2. Neighbor Generation

In the Neighbor Generation phase of Simulated Annealing (SA) task scheduling, the focus is on generating new candidate solutions by making small, incremental changes to the current schedule. This iterative process allows the algorithm to explore the solution space in a controlled manner, gradually improving the task-to-node assignments over time. The concept is to shake up the current schedule to explore the solution space and move towards the optimal or near-optimal solutions.

In each round *k*, the system picks a task $i^{*}$ at random from the set of tasks {1,…,T}. After choosing task $i^{*}$, the system gives it to a new node $j^{*}$ which is not the same as its current node $\sigma (i^{*})$. The new node is chosen randomly from the set of available nodes {1,…,N} excluding the current node assignment, i.e., $j^{*}\in \left\{1,\ldots ,N\right\}\setminus \left\{\sigma \left(i^{*}\right)\right\}$. The new schedule, $\sigma^{\prime }$, is then formed by applying this perturbation. Formally, the new schedule is given by:

(14) $$\sigma^{\prime }\left(i\right)=\left\{\begin{array}{cc} j^{*}, & \mathrm{if}\;i=i^{*},\\ \sigma (i), & \mathrm{if}\;i\neq i^{*}. \end{array}\right.$$

This simple yet effective step creates a neighboring schedule that differs slightly from the current one by ensuring that only one task is changed at a time. The algorithm ensures that the search process is controlled and focused by making only small changes to the schedule. What makes this strategy beautiful is one change to the task-node allocation affects only two workloads: the workload of the old node that performed the task, $W_{\sigma (i^{*})}$ and the workload of the new node, $W_{j^{*}}$. This small change makes it less expensive to examine the new schedule because only the two workloads that need to be updated are changed. This strategy is useful because the new schedule cost is updated incrementally. Because only two nodes are changed, the algorithm does not need to recalculate the entire schedule’s cost. It can compute the cost through the changes based on the workload nodes, which is faster and more effective. We define the cost function as Cost(σ),) where σ represents a particular schedule. After we reassign task $i^{*}$ to node $j^{*}$, we can express the change in cost ΔCost like this:

(15) $$\Delta \mathrm{Cost}=\mathrm{Cost}\left(\sigma^{\prime }\right)-\mathrm{Cost}\left(\sigma \right)=\Delta W_{\sigma (i^{*})}+\Delta W_{j^{*}}$$

where $\Delta W_{\sigma (i^{*})}$ and $\Delta W_{j^{*}}$ represent the changes in the workloads of the affected nodes. This formulation allows for efficient cost evaluation without needing to recompute the entire schedule’s cost from scratch.

A detailed search enables Simulated Annealing to methodically navigate through the solution space, tweaking small elements that impact the overall schedule cost, which is crucial in optimizing the schedule. The method guarantees that the solution space is approached in a very constrained, incremental way to avoid large, sudden shifts that may result in a locally optimal solution. Also, the changes make sure that each new schedule links to the one before, creating a chain of better and better schedules as the algorithm goes on. This method is good because it can balance looking around the solution space with using the best solutions found so far. At the beginning of the search, when the temperature is still high, the algorithm is more likely to accept steps down the hierarchy that are far from optimal in order to circumvent local optima. When the temperature is lowered, the algorithm becomes more focused on the best solutions found so far and only attempts to change them in a more fine-grained manner. The Simulated Annealing algorithm’s effectiveness in solving the task scheduling problems in the changing balance between exploration and exploitation.

Thus, the way in which neighbors are formed is critical to the SA task-scheduling algorithm. It offers a means to enhance the schedule without excessive computational expense. The algorithm is able to gradually and purposely improve the schedule by making small changes, computing the cost of each change and iteratively refining the schedule towards an optimal solution.

### 4.3. Cost Evaluation

In the Cost Evaluation phase of Simulated Annealing (SA) for task scheduling, the goal is to assess how the new schedule compares to the current one by calculating the change in cost:

(16) $$\Delta \mathrm{Cost}=\mathrm{Cost}\left(\sigma^{\prime }\right)-\mathrm{Cost}\left(\sigma \right)$$

However, instead of figuring out the whole cost from the beginning, we use the fact that one task’s assignment changes. This means we can work out the change in cost bit by bit, looking at just the parts affected by the switch. This makes our calculations more efficient.

When it comes to task scheduling, the cost function for a specific schedule σ has two main parts: the makespan and the priority penalty terms. The makespan is the total time it takes to finish all tasks, which depends on how much work each node has to do. The priority penalty term deals with the order in which tasks need to be done punishing schedules that do not follow the right task order.

For a given schedule σ, you can figure out how much work node *j* has by adding up the time it takes to do each task *i* that is assigned to that node:

(17) $$W_{j}=\sum_{i:\sigma (i)=j}d_{i}$$

Now, when we perturb the schedule by moving task $i^{*}$ from node $a=\sigma (i^{*})$ to a new node $b=j^{*}$, the workloads of nodes *a* and *b* are updated accordingly. Specifically, the new workload for node *a*, denoted $W_{a}^{\prime }$, is obtained by subtracting the duration of the moved task $d_{i^{*}}$, while the new workload for node *b*, denoted $W_{b}^{\prime }$, is obtained by adding the duration of the moved task $d_{i^{*}}$:

(18) $$W_{a}^{\prime }=W_{a}-d_{i^{*}},\;W_{b}^{\prime }=W_{b}+d_{i^{*}}$$

The changes to the node workloads have an impact on the schedule’s makespan. We define the makespan as the highest workload among all nodes. The difference between the new and old makespan is what we call the change in makespan, ΔM. The new makespan is calculated as:

(19) $$\Delta M=\max\left(W_{a}^{\prime },W_{b}^{\prime },\ldots \right)-\max\left(W_{a},W_{b},\ldots \right)$$

This equation reflects the change in the overall task completion time as a result of reassigning task $i^{*}$ to node *b*. By only recalculating the workloads of the two affected nodes, this process is computationally efficient and avoids the need to recompute the makespan for the entire schedule.

The task reassignment affects not only the makespan but also the priority-penalty term. This penalty represents the cost when tasks do not follow their priority rules. For task $i^{*}$, if it is assigned to node *a*, it incurs a priority penalty $p_{i^{*}}W_{a}$. When it is moved to node *b*, the priority penalty becomes $p_{i^{*}}\left(W_{b}+d_{i^{*}}\right)$. The change in the priority penalty, denoted ΔP, is given by:

(20) $$\Delta P=p_{i^{*}}\left(W_{b}+d_{i^{*}}\right)-p_{i^{*}}W_{a}=p_{i^{*}}\left(W_{b}+d_{i^{*}}-W_{a}\right)$$

This term adjusts the total cost based on how the task reassignment impacts the priority order and the penalty associated with violating the priority constraints.

Finally, the total change in cost Δ is a weighted sum of the changes in makespan and priority penalty:

(21) Δ=αΔM+δΔP

where α and δ are user-defined constants that control the relative importance of the makespan and priority-penalty components in the overall cost function. The values of α and δ are typically set based on the specific requirements of the scheduling problem, such as whether the makespan or priority penalties should be more heavily penalized.

Reviewing the cost changes step by step makes the Cost Evaluation process quicker. This method examines small changes that happen when tasks are moved around. It lets the system decide if the change makes things better or worse and whether to keep or reject the new plan. Fast checks are important to how Simulated Annealing works. These checks help it test many options without using too much time.

The approach works better when the cost or energy function is made simpler. If nodes behave by using resources or dealing with costs, it can balance the energy and money used. This simplifies the evaluation process and reduces its difficulty. It offers an easy way to assess costs and keeps the SA task scheduling method effective and simple to handle. This makes it a solid and flexible choice to manage scheduling for large tasks.

### 4.4. Acceptance Criterion

In the Acceptance Criterion step of Simulated Annealing, which is applied in task scheduling, deciding whether to accept a new neighbor schedule $\sigma^{\prime }$ relies on the Metropolis rule. This rule uses probability to introduce randomness into the exploration of solutions. In the annealing process, this randomness helps the algorithm look at different parts of the solution space. During this stage, the algorithm prioritizes exploring new options over refining existing ones. Using this approach prevents the algorithm from getting stuck in local optima too, ensuring it has a chance to find the best overall solution. The Metropolis rule’s core idea compares the difference in the cost function written as $\Delta =\mathrm{Cos}t\left(\sigma^{\prime }\right)-\mathrm{Cos}t\left(\sigma \right)$ to a factor that depends on temperature.

The basic form of the Metropolis rule involves comparing the change in the cost function, $\Delta =\mathrm{Cos}t\left(\sigma^{\prime }\right)-\mathrm{Cos}t\left(\sigma \right)$, with a temperature-dependent factor. If the change in cost Δ is less than or equal to zero, the proposed solution $\sigma^{\prime }$ is accepted unconditionally, as it improves the overall cost or does not worsen it. In mathematical terms, this is expressed as:

(22) $$P_{\mathrm{accept}}=\left\{\begin{array}{ll} \;1, & \mathrm{if}\;\Delta \leq 0,\\ \exp\left(\frac{-\Delta }{T_{k}}\right), & \mathrm{if}\;\Delta >0. \end{array}\right.$$

Here, $P_{\mathrm{accept}}$ represents the probability of accepting the new schedule $\sigma^{\prime }$, and $T_{k}$ is the current temperature at iteration *k*. The temperature $T_{k}$ plays a crucial role in determining the likelihood of accepting a worsening solution (i.e., a solution with Δ>0). As the temperature decreases over time (due to the cooling schedule), the acceptance probability for worse solutions becomes lower, which ensures that the algorithm gradually shifts from exploration to exploitation.

When Δ>0, meaning the new schedule $\sigma^{\prime }$ results in a worse solution, the decision to accept it is made probabilistically. The probability of accepting a worse solution decreases as the temperature lowers, with probability given by the exponential function $\exp\left(\frac{-\Delta }{T_{k}}\right)$. This function means that when the temperature is high, even large increases in cost may be accepted, allowing the algorithm to escape from local minima and explore broader regions of the solution space. On the other hand, as the temperature cools, the acceptance of worse solutions becomes increasingly rare, and the algorithm focuses more on refining the current best solution.

The acceptance decision is made by drawing a uniform random number *u* from the interval (0,1), i.e., u∼U(0,1). If this random value is less than or equal to $P_{\mathrm{accept}}$, the new schedule $\sigma^{\prime }$ is accepted, and the current schedule σ is updated to $\sigma^{\prime }$. In other words, the acceptance condition is:

(23) $$\mathrm{accept}\;\mathrm{if}\;u\leq P_{\mathrm{accept}}$$

This random acceptance mechanism ensures that an algorithm does not get stuck in local optima too early in the process. By allowing uphill moves (i.e., accepting worse solutions) with a certain probability, the algorithm maintains the capacity to explore the solution space more widely in the early stages of the process.

The temperature $T_{k}$ starts high and gradually decreases according to the cooling schedule. The annealing process begins with the algorithm choosing weaker solutions since the temperature starts off high. This allows it to explore more options. By doing so, it avoids getting stuck in poor local spots and improves its chances of reaching an optimal or global result. When the temperature lowers, the algorithm leans more toward the stronger solutions it has already uncovered. It fine-tunes its focus to move closer to the best possible outcome.

The acceptance criterion plays a key role in balancing exploration and exploitation. The temperature schedule serves as the main factor in this process. It decides how the algorithm shifts from accepting a wider range of exploratory solutions to favoring fewer, more-refined exploitative solutions. Striking this balance is crucial to allowing the Simulated Annealing algorithm to find effective solutions to tough optimization tasks like task scheduling.

### 4.5. Cooling

The Cooling and Termination phase in Simulated Annealing (SA) allows the algorithm to change from exploring possible solutions to focusing on refining a single solution. It balances searching the solution space while settling on a high-quality result. A geometric cooling schedule controls this phase by lowering the temperature $T_{k}$ with each step. The temperature update rule is given by:

(24) $$T_{k+1}=\tau T_{k}$$

where $T_{k}$ is the temperature at iteration *k*, $T_{k+1}$ is the temperature at the next iteration, and τ is a cooling rate parameter, typically chosen to lie within the range [0.90,0.99]. This cooling rate parameter τ controls the speed at which the temperature decreases. When τ is close to 1, the temperature decreases slowly, allowing the algorithm to continue exploring for a longer period of time. Conversely, smaller values of τ result in faster cooling, causing the algorithm to shift more quickly toward exploitation of the best solutions found.

The cooling schedule plays a role in balancing exploration and exploitation in the algorithm when the temperature remains high, and the acceptance rule allows it to accept worse solutions more often. This helps to explore the solution space and lets the algorithm move past local minima. When the temperature lowers, the chances of choosing weaker solutions decrease, and the algorithm focuses more on using the best solutions it has found up to that point. This gradual cooling helps the algorithm settle into an ideal or almost ideal solution while keeping the search area open enough to avoid cutting off possibilities too soon. The algorithm runs by adjusting the temperature and evaluating potential solutions. It stops when one of two conditions is met. The first condition happens if the temperature $T_{k}$ falls below a set minimum $T_{\min}$. The second condition occurs if it completes the maximum allowed number of iterations, K. The $T_{\min}$ level is typically a very low value close to zero. This ensures the process ends when there is little chance of finding further improvements. The iteration limit K is a second stopping criterion that avoids the algorithm running infinitely, setting an upper limit on the iterations and guaranteeing that the algorithm will terminate after a reasonable amount of computational effort.

As termination is reached, the algorithm outputs the optimum schedule discovered in all iterations. The schedule assigns tasks to nodes in a way that lowers the cost function or meets other optimization goals specific to the problem being solved. The best schedule balances exploration and exploitation during the annealing steps.

The way the cooling and stopping methods are managed has a big effect on the performance of the Simulated Annealing algorithm. By reducing the temperature geometrically, the algorithm ensures that it explores broadly in the early stages and converges more tightly toward a high-quality solution in the later stages. Additionally, the termination conditions provide a mechanism to stop the algorithm after a predefined amount of time or computational resources, ensuring that the solution process is both efficient and effective. The final schedule returned is the result of a delicate interplay between randomness and control, balancing the need for global exploration with the goal of achieving a high-quality, optimal schedule.

The proposed SA-based scheduling framework directly addresses the gaps identified in existing literature. While traditional metaheuristics such as ACO and PSO [20] mainly optimize makespan or energy, they do not effectively handle task priority. Similarly, reinforcement learning-based methods [12,13] improve adaptability but introduce high computational overheads. Other works, such as I-FASC [17] and EEIoMT [19], consider load balancing or energy but neglect reliability and urgency. In contrast, our model integrates four critical objectives—makespan, energy, cost, and reliability—along with a priority-aware penalty function. This explicit inclusion of task priority ensures responsiveness for urgent IoT applications, thereby positioning our method as a novel, multi-objective, and priority-aware solution for fog-enabled IoT environments.

## 5. Experimental Results

In this section, we discuss the simulation and results of the proposed Simulated Annealing (SA)-based Task Scheduling Framework. The complete proposed work carried out using Simpy simulation of benchmark datasets obtained from Google Cloud Job Workloads, representing small, medium, and large datasets [30].

Table 2 describes the simulation parameters used for our study. total no. of tasks 100–1000 with varying length between 15,000 to 900,000 considered. The simulation environment consists of 10 fog nodes and 6 virtual machines (VMs) to emulate realistic multi-core resource availability. Each VM supports a bandwidth of 200 MBPS, ensuring sufficient data transfer capability for task execution. The simulation was conducted on a Windows 11 operating system running on an Intel i7 processor, with the model implemented in SimPy.

### 5.1. Makespan

**Figure 4:**
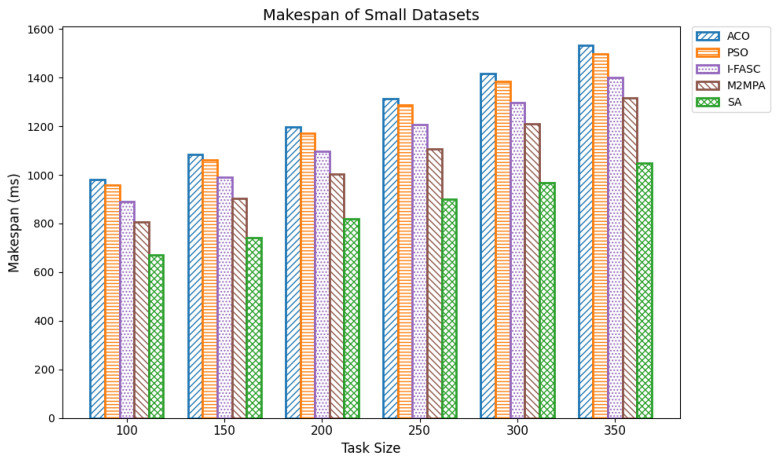
Makespan of small datasets.

**Figure 5:**
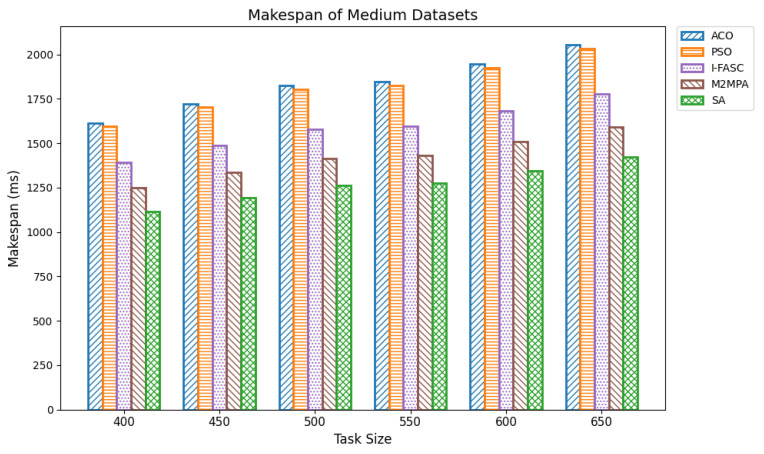
Makespan of medium datasets.

**Figure 6:**
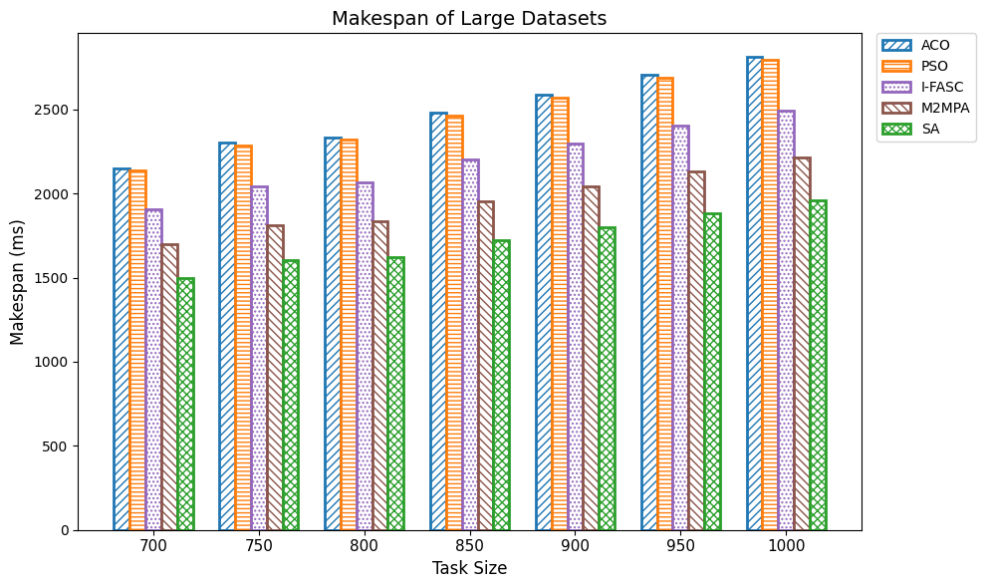
Makespan of large datasets.

In fog computing, makespan is the total time needed to complete a set of tasks in a decentralized network. It directly influences overall system effectiveness. An optimal scheduler reduces makespan by maximizing effective offloading, balancing loads across nodes, and adapting dynamically to queue and dependency conditions thereby eliminating idle time, honoring task precedences, and improving responsiveness and throughput near the edge. We evaluated makespan across varying task volumes using the Google Cloud Job Workloads benchmark. Datasets were grouped as small (100–350 tasks), medium (400–650), and large (700–1000), with task execution times ranging from 15,000 to 900,000 units. The proposed Simulated Annealing (SA) scheduler was compared against classical baselines Ant Colony Optimization (ACO) and Particle Swarm Optimization (PSO), and two stronger, recently proposed metaheuristics tailored for fog/edge settings: I-FASC (Improved Fireworks Algorithm–based Scheduling and Clustering) and M2MPA (Modified Marine Predators Algorithm). Small datasets: Table 3 (visualized in Figure 4) reports makespan for 100–350 tasks. SA consistently achieves the lowest makespan, e.g., 670.83, 742.08, 820.78, 901.56, 969.22, 1048.32 outperforming all baselines. Among the competitors, M2MPA is the strongest challenger, followed by I-FASC, then PSO, and finally ACO. This ranking indicates that even against more recent metaheuristics, SA delivers the most efficient scheduling under constrained edge resources. Medium datasets: Table 4 (Figure 5) shows the same pattern for 400–650 tasks. SA records makespans of 1115.64, 1191.61, 1263.31, 1277.57, 1346.38, and 1421.81, again beating all four baselines. As task volume grows, the gap between SA and legacy baselines (ACO/PSO) widens, while the SA vs. M2MPA/I-FASC margins remain favorable, highlighting SA’s scalability and robustness at higher loads. Large datasets: Table 5 (Figure 6) summarizes results for 700–1000 tasks. SA maintains the lead with makespans 1495.40, 1601.41, 1622.68, 1725.43, 1800.86, 1882.23, 1957.13. M2MPA remains the closest competitor, then I-FASC, with PSO and ACO trailing. SA’s advantage at scale is attributable to its ability to explore globally early on and converge adaptively, avoiding local minima and allocating work so that high-priority/long tasks are mapped to less-loaded nodes.

### 5.2. Energy Consumption

**Figure 7:**
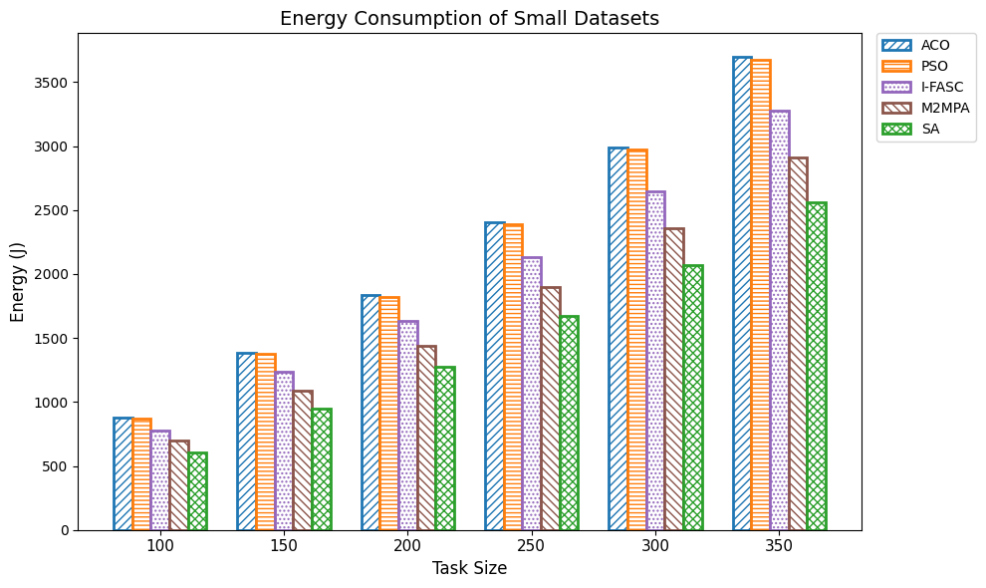
Energy consumption of small datasets.

**Figure 8:**
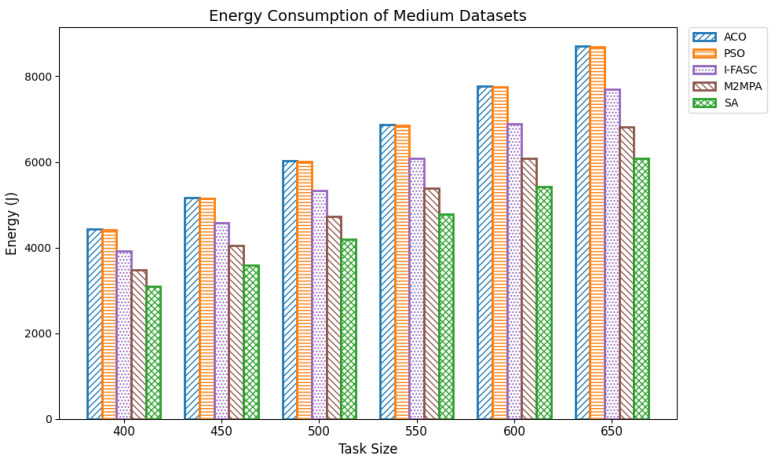
Energy consumption of medium datasets.

**Figure 9:**
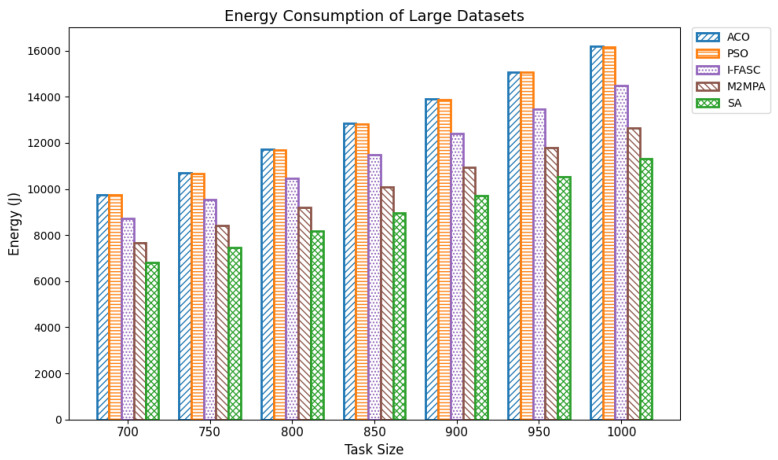
Energy consumption of large datasets.

In fog computing environments, energy consumption is a critical performance metric, particularly in edge-based systems where computational nodes often operate with limited power resources. Efficient task scheduling not only improves execution performance but also significantly reduces the total energy consumed during task execution. Lowering energy usage is vital for extending the operational lifespan of fog nodes, especially those powered by batteries or deployed in remote and resource-constrained locations. Optimized scheduling contributes by minimizing execution times, balancing computational loads, and preventing the overuse of specific nodes that would otherwise drain energy disproportionately. For our experiments, energy consumption was computed as the cumulative energy drawn by all nodes, assuming a uniform per-node power rate, with execution durations derived from the same Google Cloud Job Workload datasets used in the makespan evaluation. Datasets were grouped as small (100–350 tasks), medium (400–650 tasks), and large (700–1000 tasks). The proposed Simulated Annealing (SA) approach was compared not only with classical baselines ACO and PSO but also with two recent swarm/metaheuristic models tailored to fog systems I-FASC and M2MPA. Small datasets: Table 6 and Figure 7 show results for 100–350 tasks. SA achieves the lowest energy consumption across all task volumes, e.g., 607.34 J, 951.77 J, 1272.90 J, 1675.60 J, 2070.52 J, and 2560.49 J. M2MPA consistently comes second, followed by I-FASC, while ACO and PSO consume the most, showing a clear descending order. Medium datasets: Table 7 and Figure 8 present results for 400–650 tasks. SA again delivers the lowest values, e.g., 3091.08 J to 6074.64 J, across the range, maintaining a strong gap from ACO/PSO and a consistent advantage over I-FASC and M2MPA. Although all algorithms show proportional growth in energy usage with task size, SA’s energy footprint increases at a slower rate, highlighting its scalability. Large datasets: Table 8 and Figure 9 summarize results for 700–1000 tasks. SA records 6809.73 J, 7467.54 J, 8176.36 J, 8974.15 J, 9704.25 J, 10544.25 J, and 11319.15 J. Even in large-scale settings, SA outperforms M2MPA, I-FASC, PSO, and ACO. M2MPA again proves competitive, but SA’s ability to intelligently allocate tasks to less congested nodes and avoid hotspots leads to the most efficient energy use at scale.

### 5.3. Cost

**Figure 10:**
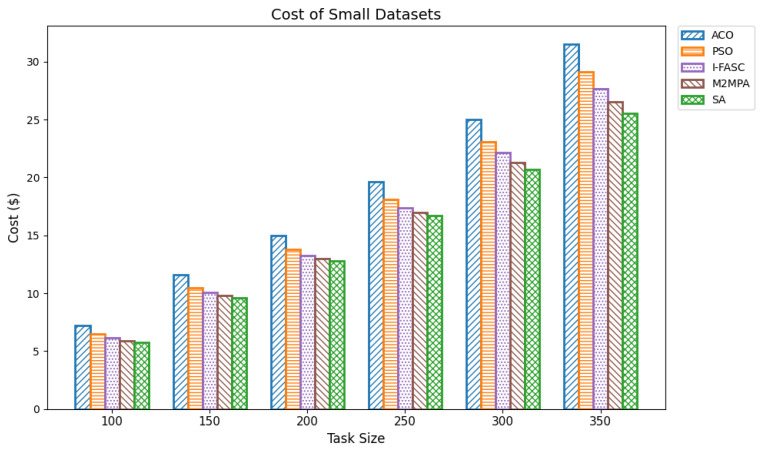
Cost of small datasets.

**Figure 11:**
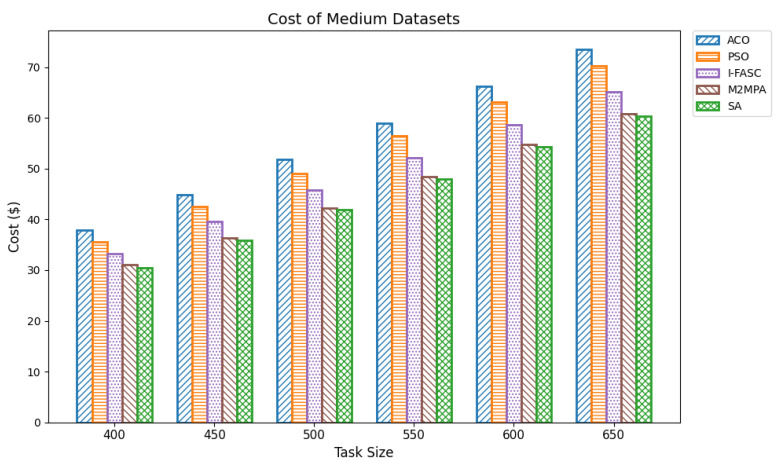
Cost of medium datasets.

**Figure 12:**
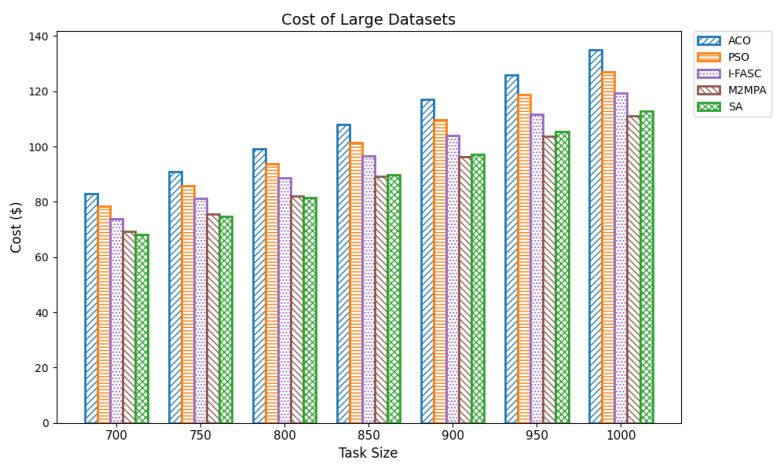
Cost of large datasets.

In fog computing infrastructures, execution cost is a vital performance indicator that reflects the economic feasibility of a scheduling strategy. Each task incurs a cost proportional to its execution time and the computational resources consumed. Since fog nodes often operate as part of commercial edge-cloud ecosystems or resource-leased platforms, minimizing cost is crucial for cost-effective and scalable deployments. An efficient scheduler reduces cost by minimizing task durations, avoiding overloading expensive nodes, and exploiting resource-efficient execution paths. In our experiments, cost was computed as the product of task execution time and a constant per-time-unit processing rate. The evaluation used the same workload datasets as for makespan and energy: small (100–350 tasks), medium (400–650 tasks), and large (700–1000 tasks). The proposed Simulated Annealing (SA) scheduler was benchmarked against classical baselines (ACO, PSO) and the more advanced metaheuristics I-FASC and M2MPA. Small datasets: Table 9 and Figure 10 summarize results for 100–350 tasks. SA achieved the lowest costs of 5.74, 9.58, 12.80, 16.68, 20.66, and 25.54, outperforming all competitors. M2MPA emerges as the second-best, followed by I-FASC, PSO, and ACO. The clear hierarchy demonstrates SA’s strong ability to reduce resource billing time by minimizing makespan and balancing node utilization. Medium datasets. Table 10 and Figure 11 show results for 400–650 tasks. SA maintains its advantage, with cost values from 30.53 to 60.32, while M2MPA and I-FASC record higher but still competitive results. ACO and PSO incur the highest costs. The controlled rise of SA’s costs with increasing workload size highlights its economic scalability and efficiency in handling larger deployments without proportionally increasing overhead. Large datasets (Table 11 and Figure 12) report costs for 700–1000 tasks. SA again dominates, with values of 68.07, 74.61, 81.64, 89.83, 97.25, 105.51, 112.90 across task sizes. Even under heavy load, SA remains consistently below M2MPA, I-FASC, PSO, and ACO. The margin of improvement at scale demonstrates SA’s suitability for economically constrained fog deployments, where cost minimization is paramount. Overall: The comprehensive analysis across Table 9, Table 10 and Table 11 and their figures establishes a consistent pattern (lower is better). This validates that the priority-aware SA scheduler not only reduces makespan and energy but also delivers superior cost-efficiency, making it a robust solution for sustainable fog and IoT scheduling.

### 5.4. Reliability

**Figure 13:**
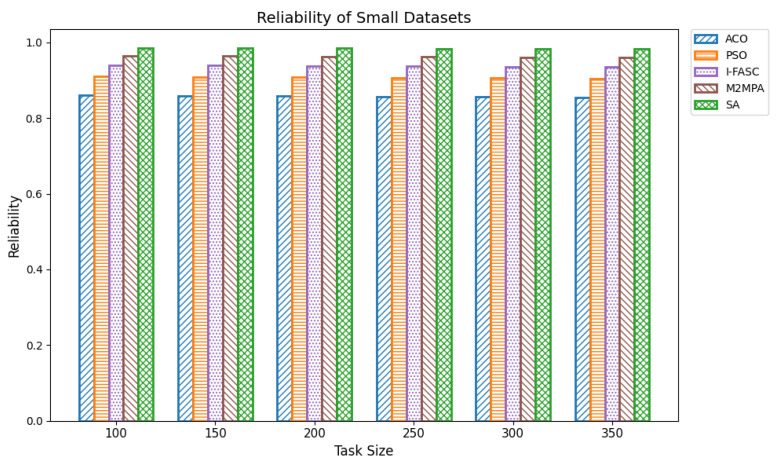
Reliability of small datasets.

**Figure 14:**
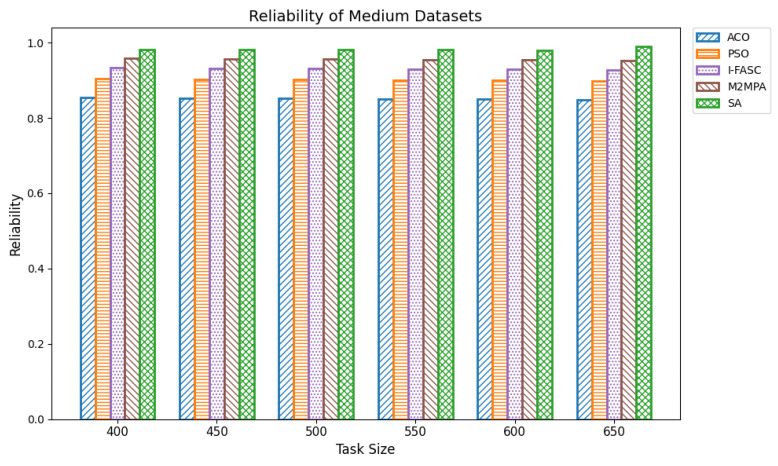
Reliability of medium datasets.

**Figure 15:**
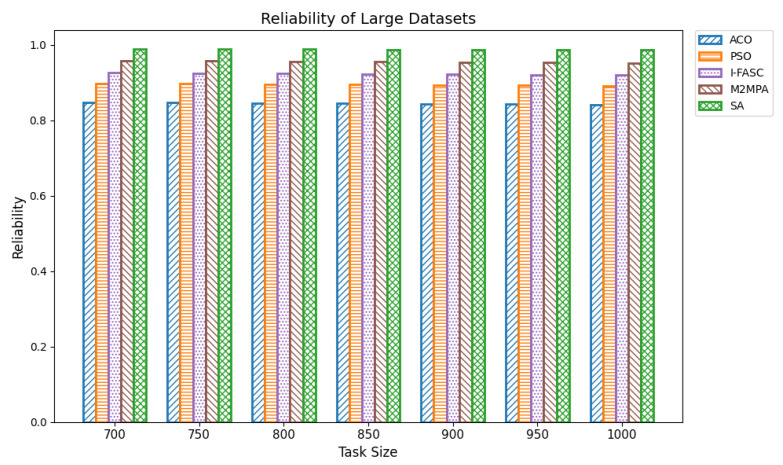
Reliability of large datasets.

In fog computing systems, reliability represents the probability that scheduled tasks are successfully executed without node failure. Given the decentralized and heterogeneous nature of fog environments, ensuring high reliability is essential, especially in mission-critical applications such as healthcare monitoring, industrial automation, and autonomous systems. Reliability in scheduling directly affects system robustness and its ability to maintain service continuity under conditions like node overload, performance degradation, or partial outages. In this work, system reliability was modeled as the compound probability that all scheduled tasks execute successfully, assuming a constant reliability factor per node. The experiments were conducted across datasets of increasing size: small (100–350 tasks), medium (400–650 tasks), and large (700–1000 tasks). The proposed Simulated Annealing (SA) scheduler was compared not only with ACO and PSO but also with two advanced metaheuristics designed for fog systems: I-FASC and M2MPA. Small datasets: Table 12 and Figure 13 show the reliability outcomes for 100–350 tasks. SA consistently achieved the highest reliability, with values of 0.9849, 0.9846, 0.9842, 0.9834, 0.9830, and 0.9823 across increasing task counts. M2MPA follows closely, then I-FASC, while PSO and ACO trail behind. The results demonstrate that SA not only balances workloads effectively but also avoids risky node assignments, ensuring higher task completion guarantees. Medium datasets: Table 13 and Figure 14 extend the analysis to 400–650 tasks. SA achieved reliability values of 0.9818, 0.9817, 0.9813, 0.9805, 0.9802, and 0.9896, consistently outperforming all baselines. M2MPA and I-FASC remain competitive, but SA’s adaptive search and dynamic task reassignment strategies maintain a clear edge. As workloads scale up, SA continues to prevent node saturation, sustaining reliability above competing models. Large datasets: Table 14 and Figure 15 summarize reliability results for 700–1000 tasks. SA again demonstrates the highest dependability with scores of 0.9889, 0.9886, 0.9883, 0.9875, 0.9873, 0.9860, and 0.9862. Even under heavy load, SA ensures stable and robust performance. M2MPA comes second, then I-FASC, followed by PSO and ACO. The superiority of SA here underscores its ability to sustain reliability at scale, making it suitable for environments where task failures are unacceptable.

The comparison algorithms ACO and PSO are metaheuristic approaches, but they often suffer from premature convergence and lack of ability to adapt dynamic fog–cloud workloads, which reduces their applicability in latency-sensitive environments. Our SA-based method addresses these challenges through a local neighborhood search with priority-aware penalties, which helps in handling urgent tasks more effectively. Frame works like I-FASC and M2MPA are important fog scheduling, but their focus is mainly on cost efficiency and throughput, and they do not address the priority handling or urgent task responsiveness. By integrating urgency and priority awareness into the scheduling process, our SA method goes beyond these frameworks. Therefore, ACO, PSO, I-FASC, and M2MPA are used not only as benchmarks but also as examples of existing limitations that our proposed solution directly overcomes.

For evaluation, we compared our proposed SA-based framework against ACO, PSO, I-FASC and M2MPA. These methods were selected as baselines because they are widely used metaheuristics in fog and cloud scheduling [14,20,26,27]. The comparison was carried out under identical experimental conditions using the Google Cloud Job Workloads dataset, with the same number of tasks, fog nodes, and bandwidth constraints. Performance was measured on makespan, energy consumption, cost, and reliability. This ensures that improvements observed with SA are attributable to the proposed framework rather than differences in setup. Furthermore, while ACO and PSO optimize scheduling, they do not incorporate task priority; our SA-based model uniquely integrates a priority-aware penalty function, providing an additional justification for its effectiveness.

## 6. Conclusions

In this work, we propose a priority-sensitive, multi-objective task scheduling framework for fog computing environments based on the simulated annealing (SA) algorithm. By incorporating a task-priority penalty function to formulate the task scheduling problem as a compound optimization challenge with improved makespan, energy consumption, execution cost, and reliability, we address the fundamental weakness of current static and single-objective methods. The proposed model shows flexibility for the dynamic and heterogeneous nature of fog infrastructures, effectively balancing computational loads while guaranteeing timely execution of high-priority tasks. Our SA-based method efficiently traverses the enormous scheduling solution space by adopting a probabilistic exploration–exploitation strategy and achieves near-optimal task-node mappings even in large-scale settings. Large-scale experimental results on various task volumes validate the proposed model as it outperforms proven metaheuristics such as ACO, PSO, I-FASC and M2MPA on all major performance measures such as makespan minimization, energy efficiency, cost-effectiveness, and reliability improvement. The framework’s ability to dynamically adapt to task urgency and responsive changes in resource availability positions it as an effective solution for latency-aware, mission-critical applications in fog-based IoT environments. Future work will include the integration of real-time feedback mechanisms, improving fault-tolerance in heterogeneous and mobile fog architectures, and integrating learning-based metaheuristics to further improve scheduling efficiency under uncertain and dynamic workload conditions.

## Figures and Tables

**Table 1:** Parameter study of various existing works.

S.No	Study/Algorithm	Metrics	Tool/Platform Used
[12]	Fuzzy Reinforcement Learning (FRL) + MNILP	Makespan, Deadline Satisfied Tasks, Average Service Time, CPU Utilization, Average Computation Time, Energy Consumption	Python-based tool
[13]	GA, SARSA, Q-Learning	Throughput, Latency, Makespan	MATLAB
[14]	MoDGWA	Makespan, Scheduling Failure Factor, Monetary Cost, Total Cost Score	MATLAB
[15]	FIS + Genetic Algorithm (GA)	Makespan, Resource Utilization, Cost	WorkflowSim
[16]	M2MPA	Makespan, Energy Consumption	-
[17]	I-FASC	Task Processing Time, Load Balancing	Alibaba Cloud Server, Core i5, 4GB RAM
[18]	FLight	Training Time Efficiency, Accuracy	-
[19]	EEIoMT	Network Usage, Latency, Energy Consumption, CPU Usage	iFogSim2
[20]	TSSAC	Energy Consumption, Makespan, Load Balancing	-
[21]	EDLB	Makespan, Resource Utilization, Load Balancing, Cost	-
[22]	DQN (Deep Q-Network)	Latency, Execution Time, Accuracy, Energy Consumption, Throughput	Cooja
[23]	ReLIEF	Reliability, Workload Distribution, Throughput, Delay	Real Time
[24]	ACO + PSO	Response Time, Load balancing and Standard deviation	MATLAB
[25]	PSO-WOA	execution time, cost, and energy	Workflowsim
[26]	Adaptive Priority Task Scheduling	makespan, failure rate, and execution delay	iFogSim
[27]	RAPTS	Resource Utilization, Response Time, Makespan, Deadline, and Cost	iFogSim
[28]	NCARO	service time, cost, and energy consumption, makespan	MATLAB
[29]	FODAS	Deadline, makespan, and energy consumption	COSCO

**Table 2:** Simulation parameters.

Simulation Parameters	Details
No. of Tasks	100–1000 tasks
Lengths of the tasks	15,000–900,000
Fog Nodes	10
VMs per node	6
Bandwidth	200 MBPS
Operating System	Windows
Processor	Intel i7
Simulation Tool	SimPy

**Table 3:** Makespan of small datasets.

Datasets	ACO	PSO	I-FASC	M2MPA	SA
100 tasks	981.21	958.33	890.45	805.62	670.83
150 tasks	1085.40	1060.11	990.38	902.14	742.08
200 tasks	1198.62	1172.54	1095.72	1004.35	820.78
250 tasks	1313.77	1287.94	1206.44	1108.29	901.56
300 tasks	1415.92	1384.60	1298.55	1210.71	969.22
350 tasks	1532.85	1497.60	1402.18	1315.86	1048.32

**Table 4:** Makespan of medium datasets.

Datasets	ACO	PSO	I-FASC	M2MPA	SA
400 tasks	1612.33	1593.77	1394.55	1249.52	1115.64
450 tasks	1719.62	1702.31	1489.51	1334.60	1191.61
500 tasks	1824.85	1804.73	1579.14	1414.91	1263.31
550 tasks	1845.98	1825.11	1596.96	1430.88	1277.57
600 tasks	1945.26	1923.41	1682.98	1507.95	1346.38
650 tasks	2054.30	2031.16	1777.26	1592.43	1421.81

**Table 5:** Makespan of large datasets.

Datasets	ACO	PSO	I-FASC	M2MPA	SA
700 tasks	2151.84	2136.29	1908.12	1697.34	1495.40
750 tasks	2302.92	2288.01	2041.67	1810.92	1601.41
800 tasks	2334.17	2318.26	2068.45	1833.56	1622.68
850 tasks	2481.94	2464.91	2204.33	1954.28	1725.43
900 tasks	2589.81	2572.66	2297.58	2039.76	1800.86
950 tasks	2706.68	2688.90	2402.15	2130.64	1882.23
1000 tasks	2812.55	2795.91	2494.20	2215.36	1957.13

**Table 6:** Energy consumption of small datasets.

Datasets	ACO	PSO	I-FASC	M2MPA	SA
100 tasks	878.45	867.12	780.36	695.84	607.34
150 tasks	1386.74	1373.19	1235.25	1090.46	951.77
200 tasks	1832.58	1818.43	1632.41	1440.55	1272.90
250 tasks	2407.21	2391.29	2130.84	1896.72	1675.60
300 tasks	2990.80	2972.17	2649.12	2358.96	2070.52
350 tasks	3697.45	3677.84	3276.42	2912.88	2560.49

**Table 7:** Energy consumption of medium datasets.

Datasets	ACO	PSO	I-FASC	M2MPA	SA
400 tasks	4431.90	4417.26	3925.44	3480.27	3091.08
450 tasks	5170.31	5154.20	4574.38	4056.61	3597.74
500 tasks	6031.55	6016.37	5338.42	4728.66	4201.38
550 tasks	6863.93	6846.55	6081.15	5380.42	4782.54
600 tasks	7774.98	7756.87	6882.77	6089.62	5425.81
650 tasks	8704.77	8685.21	7701.36	6822.43	6074.64

**Table 8:** Energy consumption of large datasets.

Datasets	ACO	PSO	I-FASC	M2MPA	SA
700 tasks	9740.88	9728.33	8705.64	7658.22	6809.73
750 tasks	10,693.75	10,681.02	9556.41	8402.15	7467.54
800 tasks	11,708.17	11,694.80	10,463.72	9204.86	8176.36
850 tasks	12,844.17	12,829.36	11,475.23	10,100.37	8974.15
900 tasks	13,894.74	13,878.21	12,405.64	10,935.72	9704.25
950 tasks	15,080.95	15,063.22	13,463.88	11,807.46	10,544.25
1000 tasks	16,188.41	16,170.07	14,471.23	12,649.38	11,319.15

**Table 9:** Cost of small datasets.

Datasets	ACO	PSO	I-FASC	M2MPA	SA
100 tasks	7.20	6.50	6.18	5.91	5.74
150 tasks	11.60	10.50	10.08	9.78	9.58
200 tasks	15.00	13.80	13.24	13.00	12.80
250 tasks	19.60	18.10	17.35	17.00	16.68
300 tasks	25.00	23.10	22.18	21.30	20.66
350 tasks	31.50	29.10	27.64	26.50	25.54

**Table 10:** Cost of medium datasets.

Datasets	ACO	PSO	I-FASC	M2MPA	SA
400 tasks	37.90	35.60	33.20	31.10	30.53
450 tasks	44.80	42.50	39.60	36.40	35.83
500 tasks	51.90	49.10	45.80	42.30	41.92
550 tasks	59.00	56.50	52.20	48.50	48.03
600 tasks	66.20	63.10	58.60	54.70	54.25
650 tasks	73.50	70.20	65.10	60.80	60.32

**Table 11:** Cost of large datasets.

Datasets	ACO	PSO	I-FASC	M2MPA	SA
700 tasks	83.00	78.50	73.90	69.20	68.07
750 tasks	90.80	85.90	81.40	75.60	74.61
800 tasks	99.20	93.70	88.80	82.10	81.64
850 tasks	108.00	101.50	96.50	89.30	89.83
900 tasks	117.00	109.80	104.10	96.30	97.25
950 tasks	126.00	118.90	111.80	103.70	105.51
1000 tasks	135.00	127.00	119.50	111.10	112.90

**Table 12:** Reliability of small datasets.

Datasets	ACO	PSO	I-FASC	M2MPA	SA
100 tasks	0.8600	0.9100	0.9400	0.9650	0.9849
150 tasks	0.8590	0.9090	0.9390	0.9640	0.9846
200 tasks	0.8580	0.9080	0.9380	0.9630	0.9842
250 tasks	0.8570	0.9070	0.9370	0.9620	0.9834
300 tasks	0.8560	0.9060	0.9360	0.9610	0.9830
350 tasks	0.8550	0.9050	0.9350	0.9600	0.9823

**Table 13:** Reliability of medium datasets.

Datasets	ACO	PSO	I-FASC	M2MPA	SA
400 tasks	0.8540	0.9040	0.9330	0.9580	0.9818
450 tasks	0.8530	0.9030	0.9320	0.9570	0.9817
500 tasks	0.8520	0.9020	0.9310	0.9560	0.9813
550 tasks	0.8510	0.9010	0.9300	0.9550	0.9805
600 tasks	0.8500	0.9000	0.9290	0.9540	0.9802
650 tasks	0.8490	0.8990	0.9280	0.9530	0.9896

**Table 14:** Reliability of large datasets.

Datasets	ACO	PSO	I-FASC	M2MPA	SA
700 tasks	0.8480	0.8980	0.9260	0.9580	0.9889
750 tasks	0.8470	0.8970	0.9250	0.9570	0.9886
800 tasks	0.8460	0.8960	0.9240	0.9560	0.9883
850 tasks	0.8450	0.8950	0.9230	0.9550	0.9875
900 tasks	0.8440	0.8940	0.9220	0.9540	0.9873
950 tasks	0.8430	0.8930	0.9210	0.9530	0.9860
1000 tasks	0.8420	0.8920	0.9200	0.9520	0.9862

## Data Availability

The original data presented in the study are openly available in a publicly accessible repository: Hussain, A.; Aleem, M. GoCJ: Google cloud jobs dataset for distributed and cloud computing 811 infrastructures. *Data* **2018**, *3*, 38. 812. https://data.mendeley.com/datasets/b7bp6xhrcd/1.
